# Arthroplasty for Treating Proximal Femur Metastatic Lesions May Be Associated with Lower Mortality Rates Compared to Intramedullary Nailing within the VA Healthcare System

**DOI:** 10.3390/jcm12175717

**Published:** 2023-09-01

**Authors:** Phillip W. Lam, David Putnam, Marissa M. Song Mayeda, Kenneth R. Gundle

**Affiliations:** 1 Department of Orthopaedics & Rehabilitation, Oregon Health & Science University, Portland, OR 97239, USA; 2 Operative Care Division, Portland VA Medical Center, Portland, OR 97239, USA

**Keywords:** metastasis, pathologic fracture, femur, arthroplasty, intramedullary nail

## Abstract

Metastatic bony disease is a significant health issue, with approximately 700,000 new cases annually that tend to metastasize to bones. The proximal femur in the appendicular skeleton is commonly affected. Our study aimed to investigate mortality rates and hospital stay duration in patients with pathologic proximal femur fractures treated with either intramedullary nailing or arthroplasty within the Veterans Health Administration system. In total, 679 patients (265 arthroplasty, 414 intramedullary nails) were identified through ICD-9 and CPT codes from 30 September 2010 to 1 October 2015. Hospital stays were similar for both groups (arthroplasty: 10.5 days, intramedullary nails: 11 days, *p* = 0.1). Mortality was associated with increased age and Gagne comorbidity scores (*p* < 0.001). Arthroplasty showed a survival benefit in the log-rank test (*p* = 0.018), and this difference persisted in the multivariate analysis after adjusting for age and comorbidities, with a hazard ratio of 1.3. Our study reported evidence that arthroplasty is associated with increased patient survival even when accounting for age and comorbidities in treating metastatic disease of the proximal femur.

## 1. Introduction

Metastatic disease affecting the skeletal system represents a large burden of disease, with approximately 700,000 new cases each year of subtypes with a propensity for bone metastasis [1]. In the appendicular skeleton, the most common site of metastatic disease is in the proximal femur, a region which is exposed to loads as high as 1200 pounds per square inch [2]. Metastatic lesions to the proximal femur significantly alter the stiffness and load to failure of this region [3], increasing the risk of a pathologic fracture.

Treatment goals for patients who sustain pathologic fractures of the proximal femur include pain relief, return to function, and the avoidance of further surgery. Historically such injuries have been treated with intramedullary nailing or a form of arthroplasty including long-stemmed hemiarthroplasty, total hip arthroplasty, or proximal femur replacement. With some authors reporting bony healing rates as low as 35% in pathologic fractures [4], implant selection becomes a critical choice as bony healing cannot be relied upon to take biomechanical stresses off the implant. Therefore, whichever implant is selected, it must have the structural integrity to last the expected lifetime of the patient in order to avoid the morbidity and mortality of revision surgery. Several authors have demonstrated lower rates of reoperation with arthroplasty [5,6,7] techniques. In addition, other studies have demonstrated higher rates of reoperation with intramedullary nailing techniques [8,9]. In 2018, our group performed a pooled meta-analysis of 16 studies and were not able to demonstrate a difference in reoperation rates between the two techniques [10].

The aim of this study was to investigate the mortality rates and duration of hospitalization among patients with pathologic proximal femur fractures who received treatment with either intramedullary nailing or arthroplasty within the Veterans Health Administration system. Arthroplasty is often considered a more invasive procedure than intramedullary nailing due to a larger incision, more soft tissue dissection, and the inability to perform the procedure percutaneously. Despite this, the literature varies regarding differences in overall survival when comparing implant choices [5,7], largely due to small sample size and confounding. We hoped to overcome these issues by using validated comorbidity scores to adjust for comorbidities and to utilize a large integrated clinical database to improve our sample size.

## 2. Materials and Methods

### 2.1. Study Design

This retrospective database comparative study included patients in the national Veterans Health Administration (VHA) with surgery taking place between 30 September 2010 and 1 October 2015. Data were then collected until the patients’ last known follow-up or death. This work was supported by resources at and the use of facilities at the Portland Veterans Administration Medical Center (Portland, OR, USA).

### 2.2. Patients

The VHA represents a large integrated healthcare system that operates throughout the United States. Multiple databases exist within this system that capture healthcare related activities. The Veterans Administration Informatics and Computing Infrastructure Corporate Data Warehouse (VINCI) is a constantly refreshed relational database that comprehensively captures data on veteran healthcare, encompassing medical services provided within and outside the VA system. Notably, this includes inpatient and outpatient procedures, clinic visits, and skilled nursing facility stays. The database includes data such as diagnoses, vitals, laboratory values, and surgical procedures which are then connected to patient identifiers, CPT, and ICD codes to allow for billing to occur. While certain data within the database serve billing purposes, its primary focus is on clinical information rather than functioning as a claims-based administrative database. This enhances the database’s utility for investigating matters like reoperation within our national cohort.

After obtaining IRB approval for this study, we accessed VINCI to define our cohort. We chose to include patients within a 5-year period from 30 September 2010 to 1 October 2015. This simplified coding for this project as these included patients prior to the implementation of ICD-10 codes. We began by querying the database to identify patients with specific ICD-9 codes indicating metastatic disease and pathologic fractures of the proximal femur, namely ICD-9 codes 733.14 (pathologic fracture, neck of femur), 733.15 (pathologic fracture, other part of femur), and 733.10 (pathologic fracture, unspecified site). Additionally, we looked for the corresponding CPT codes indicating an orthopedic hip surgery, which included the following codes: 27245 (open treatment, femur/hip with nail) and 27236 (open treatment of femoral fracture, proximal end, neck, including prosthetic replacement). We then used the database to assess patient survival.

### 2.3. Variables, Outcome Measures, and Bias

For each patient, we collected variables and demographic information including age, gender, primary cancer diagnosis, date of death or last follow-up, and comorbidities. Comorbidity data were compared between patients using the Gagne comorbidity score which is a numeric comorbidity score designed to predict short- and long-term mortality. Initially, the Gagne comorbidity score was tested in a cohort of approximately 120,000 Pennsylvania Medicare patients and then subsequently validated in a cohort of New Jersey Medicare patients. It combines aspects of both the Charlson and Elixhauser comorbidity scores and has been found to predict mortality better than either of these two indices alone [11].

## 3. Results

Our query identified 679 patients for inclusion in this study. Out of the total 679 patients, 265 individuals underwent arthroplasty (CPT 27236), while 414 received treatment with intramedullary nails (CPT 27245). As expected within the VHA population, 95% of these patients were male, with lung (24%), prostate (19%), and hematologic malignancies (15%) being the predominant cancer diagnoses. Additional malignancies included: renal (10%), head and neck (9%), gastrointestinal (8%), skin (5%), liver (3%), bladder (3%), and unknown primary (4%). There was no significant difference in the distribution of diagnoses between the two groups (*p* = 0.3) The follow-up period was longer for the arthroplasty group, lasting approximately 2.3 years, in comparison to the intramedullary nail group, which had an average follow-up of 1.9 years (*p* = 0.01). The arthroplasty cohort was also older (mean age 73 vs. 69, *p* = 0.0001) and less comorbid (Gagne 7.2 vs. 6.3, *p* = 0.003). When comparing length of stay between the two cohorts, we found that they were similar (arthroplasty 10.5 days vs. intramedullary nail 11 days, *p* = 0.1).

**Figure 1:**
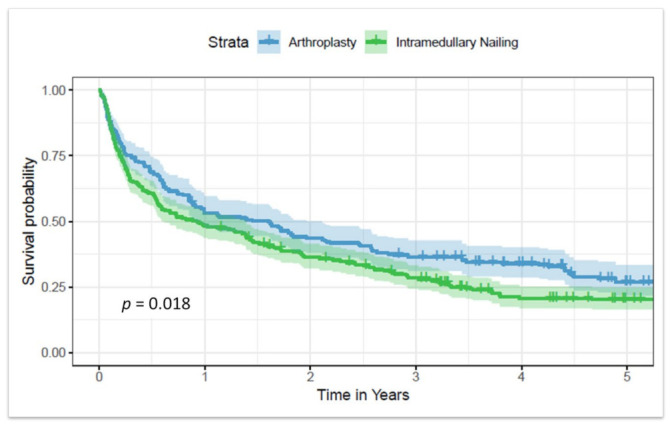
Log-rank test of patients treated with arthroplasty versus intramedullary nailing.

During our data analysis for the primary outcome of mortality, we observed that advanced age and higher Gagne comorbidity scores were linked to reduced survival (*p* < 0.001). Furthermore, arthroplasty as the treatment method for proximal femur metastatic disease showed a significant association with survival (Figure 1), as evidenced by the log-rank test results (*p* = 0.018), when compared to intramedullary nailing. After performing a multivariate analysis adjusting for age and comorbidities, this difference persisted with a hazard ratio of 1.3.

## 4. Discussion

In this retrospective cohort study utilizing a database, we observed that when arthroplasty was employed as the treatment for proximal femur metastatic disease, the results exhibited a higher patient survival rate compared to intramedullary nailing. Furthermore, this difference in survival persisted even after adjusting for age and comorbidities.

Although arthroplasty is often regarded as a more invasive procedure than intramedullary nailing due to the larger incisions and more extensive soft tissue dissection, this study demonstrated that it is associated with improved survival. However, questions remain in the literature about which implant is associated with improved survival. In 2009, Sarahrudi et al. [12] published a retrospective series of 142 patients with pathologic femur fractures related to metastatic disease and found that the use of endoprosthetic replacement was associated with decreased survival as compared to osteosynthetic constructs. They also found that overall survival was associated with tumor type, with breast cancer patients surviving longer than other cancer subtypes. Importantly, this study included patients treated with dynamic hip screws and dynamic condylar screws which were associated with high mechanical failure rates leading the authors to recommend endoprosthetic reconstruction for patients with metastatic pathologic femur fractures. Similarly, Mavrogenis et al. [13] demonstrated a significantly increased survival with intramedullary nailing as compared to resection and endoprosthetic reconstruction in a survival analysis of 110 patients with femoral metastases. In contrast, Lin et al. [7] demonstrated no difference in survival between 86 patients treated for proximal femur metastatic disease with intramedullary nails or hemiarthroplasty. Likewise, in 2011, Zacherl et al. [8] reported on a cohort of 59 patients with proximal femur metastatic disease treated with intramedullary nailing or resection and reconstruction and noted no statistical difference in the median survival (12.6 months) between groups.

Given that arthroplasty is generally associated with lower long term mechanical failure rates than intramedullary nailing [6,7,14], it is often recommended for patients with a lower burden of metastatic disease or fewer comorbidities. As a result, intramedullary nails are sometimes selected as a treatment option in patients who have a poor prognosis. We attempted to mitigate this potential source of confounding by utilizing the Gagne comorbidity score to adjust for comorbidities between groups. Adding a scoring system specific to metastatic bone disease may have allowed for further comparison between groups but would have required data that were not available. However, the Gagne score has been validated in large populations [15], and this study found that the survival differences persisted after adjustment for comorbidities.

The concept that intramedullary nailing is less invasive or less morbid than arthroplasty was further challenged by our results regarding length of stay. Although intramedullary nails typically require smaller incisions and do not involve the same amount of soft tissue resection and bony resection as arthroplasty constructs, we found that the length of stay following both procedures was not statistically significantly different. This may reflect the deconditioning that is present in patients with metastatic disease, imparting an increased need for inpatient rehabilitation and medical management following general anesthesia and musculoskeletal surgery.

Pathologic hip fractures and their care have many similarities with geriatric hip fractures. A registry study by Bliemel et al. [16] showed that walking ability, rates of hospital readmissions, and rate of reoperation were not statistically different. A key finding from this study was that the odds of passing away in the first 120 days were significantly higher in those who sustained a pathologic hip fracture (odds ratio: 3.07, *p* = 0.003).

The limitations of this study include many well-described issues associated with database studies including coding errors and the resultant low sensitivity of utilizing CPT codes to select cohorts. Also, this was a retrospective analysis of continuously collected clinical data in which we were not able to ascertain which indication was used to determine the implant choice. Given these limitations, there is a risk of unaccounted-for variables that could explain the observed differences in mortality seen between the cohorts. The sensitivity and specificity of utilizing ICD-9 and CPT codes for cohort creation is not known as a gold standard; however, this methodology has been utilized widely in the literature evaluating the care of metastatic disease and in many other sub-specialties of orthopedics [11,17]. Additional CPT codes could also be investigated in future studies, or raw operative notes could be reviewed to assess indications. It is possible that some patients underwent arthroplasty for the purposes of curative intent surgery in the setting of oligometastatic disease, and that may partially explain our findings. In the VA population, the vast majority of patients are men resulting in a low representation of women and those with breast or ovarian cancer which limits the generalizability to that population. Importantly, the VINCI database is a relatively unique entity within the United States and data collection techniques within this system likely differ from those used in other national databases. Prior work [18] has demonstrated that even when patients share similar demographics, the results may differ when submitting similar queries to different databases, and validation in other data sources would be beneficial.

Despite the above limitations, this study design is appropriate for this clinical question. As others have noted [19,20], database studies are best suited for studying rare outcomes in which the result might not be expected or the difference in outcomes might be too small to detect with the sample size seen in other study designs. Database studies are also well suited for study questions that could be influenced by system-wide practices, whether that is a single hospital, an entire country, or a large healthcare system such as the VHA. These results also support broader database investigation of arthroplasty versus nailing in the setting of metastatic disease and raise questions for prospective investigation of this important topic.

Although there are very limited data regarding the long-term follow-up after pathologic hip fracture, likely due to the increased mortality rate [16], there are certain data within the geriatric hip fracture data literature. Hashimoto et al. [21] demonstrated that the main causes of death for geriatric hip fractures were senility, aspiration pneumonia, bronchopneumonia, worsening heart failure, acute myocardial infarction, and abdominal aortic aneurysm. The leading cause of death within 1 year reported from that study was aspiration pneumonia and the leading cause of death after 1 year was senility. Our study did not capture the specific cause of death specifically, which may be of interest in future investigations. Additionally, these are palliative procedures in patients with life-limiting illnesses. The purpose is to improve pain control, quality of life, and mobility. We did not assess the involvement of palliative care teams nor the rate of patients enrolling in hospice. This may also be of interest in further research on pathologic femur fractures in the setting of metastatic bone disease.

Ultimately, implant selection for the management of proximal femur metastatic lesions should occur in the context of the whole patient, as the underlying cause for the association we observed between implant choice and survival is not clear at this time. However, if arthroplasty is selected as a treatment modality, our data provide some reassurance that mortality risk is not increased by choosing this procedure over intramedullary nailing.

## 5. Conclusions

In conclusion, this retrospective query of a national integrated database may have demonstrated improved survival when arthroplasty was utilized in the treatment of metastatic lesions of the proximal femur, and this difference persisted when adjusting for age and comorbidities.

## Data Availability

Data available on request due to restrictions. The data presented in this study are available on request from the corresponding author.
